# Low-level laser therapy for treatment of neurosensory disorders after orthognathic surgery: A systematic review of randomized clinical trials

**DOI:** 10.4317/medoral.21968

**Published:** 2017-10-21

**Authors:** Marcos-Alan-Vieira Bittencourt, Luiz-Renato Paranhos, Paulo-Ricardo-Saquete Martins-Filho

**Affiliations:** 1 Graduate Program in Dentistry, Federal University of Sergipe, Aracaju, Sergipe, Brazil; 2 School of Dentistry, Federal University of Bahia, Salvador, Bahia, Brazil; 3 Department of Dentistry, Federal University of Sergipe, Lagarto, Sergipe, Brazil; 4 Investigative Pathology Laboratory, Federal University of Sergipe, Aracaju, Sergipe, Brazil

## Abstract

**Background:**

Low-level laser has been widely used in Dentistry and many studies have focused on its application in oral surgeries. This study was conducted with the aim of searching for scientific evidence concerning the effectiveness of laser to reduce pain or paresthesia related to orthognathic surgery.

**Material and Methods:**

An electronic search was performed in PubMed, Scopus, Science Direct, LILACS, SciELO, CENTRAL, Google Scholar, OpenGrey, and ClinicalTrials.gov, up to November 2016, with no restrictions on language or year of publication. Additionally, a hand search of the reference list of the selected studies was carried out. The PICOS strategy was used to define the eligibility criteria and only randomized clinical trials were selected.

**Results:**

Out of 1,257 identified citations, three papers fulfilled the criteria and were included in the systematic review. The risk of bias was assessed according to the Cochrane Guidelines for Clinical Trials and results were exposed based on a descriptive analysis. One study showed that laser therapy was effective to reduce postoperative pain 24 hours (*P*=0.007) and 72 hours (*P*=0.007) after surgery. Other study revealed the positive effect of laser to improve neurosensory recovery 60 days after surgery, evaluated also by the two-point discrimination (*P*=0.005) and sensory (*P*=0.008) tests. The third study reported an improvement for general sensibility of 68.75% for laser group, compared with 21.43% for placebo (*P*=0.0095), six months after surgery.

**Conclusions:**

Individual studies suggested a positive effect of low-level laser therapy on reduction of postoperative pain and acceleration of improvement of paresthesia related to orthognathic surgery. However, due to the insufficient number and heterogeneity of studies, a meta-analysis evaluating the outcomes of interest was not performed, and a pragmatic recommendation about the use of laser therapy is not possible. This systematic review was conducted according to the statements of PRISMA and was registered at PROSPERO under the number CRD42016043258.

** Key words:**Low-level laser therapy, orthognathic surgery, parestesia, postoperative pain.

## Introduction

Orthognathic surgery is a useful procedure to correct dentofacial deformities. Le Fort I and bilateral sagittal split osteotomies are the most commonly surgical techniques used in orthognathic surgeries. The popularity of Le Fort I osteotomy dates from the study by Bell (1), in 1975, and the bilateral sagittal split osteotomy was first described by Trauner and Obwegeser (2), in 1957, and modified by Dal Pont (3), in 1961, Hunsuck (4), in 1968, and Epker (5), in 1977, as an attempt to improve stability and reduce the potential complications of the surgical procedure.

Pain and swelling are consequences of tissue injury, and procedures such as cryotherapy and the use of drugs (analgesics and anti-inflammatories) help to control these unwanted effects. However, the use of analgesics and anti-inflammatories may lead additional side-effects including gastric or intestinal irritation, cutaneous rash, neutropenia, and hepatic and renal disorders, which may reduce their benefits (6). Besides, much research interest has focused on the injury of the inferior alveolar nerve, which runs through the lower jaw in the region of the osteotomy cuts. After the surgery, many patients experience paresthesia of the lower lip and chin, generally improved over a period of months (7-9).

These neurosensory disturbances remain a complex problem and are not always easily resolved. Recently, the therapeutic use of low-level lasers, described in the literature as producing a biomodulatory effect, has been indicated in cases of pain and tissue repair (10). To penetrate the tissue, the energy delivered through a low-intensity laser device undergoes multiple scattering, which affects its distribution. Absorption of this energy stimulates or inhibits enzymatic activities and photochemical reactions that induce cascades of reactions and physiological processes with therapeutic connotations. Then, laser mediates inflammation and activates the immune system with broad therapeutic effects (11).

Based on that, many researchers have studied the application of low-level laser therapy and its effects on the reduction of post-operative pain, acceleration of recovery, and restoration of normal function of the injured nerve (12,13). A previous meta-analysis of the literature has shown that the low-level laser therapy is an effective tool for promoting wound repair (14), but other has stated that it has just a moderate analgesic effect on the masticatory muscle or joint capsule for temporomandibular joint pain (15). As there are still many controversies surrounding the real effects of laser application in orthognathic surgery, the present systematic review aims to investigate and exposure the scientific evidence that supports the use of low-level laser in postoperative pain and paresthesia.

## Material and Methods

1. Protocol and registration

The present systematic review was conducted according to the statements of the Preferred Reporting Items for Systematic Reviews and Meta-analyses (PRISMA) (16), with guidance from the Cochrane Handbook for Systematic Reviews of Interventions (17). The systematic review protocol was registered at the International Prospective Register of Systematic Reviews (PROSPERO) under the number CRD42016043258 (http://www.crd.york.ac.uk/PROSPERO).

2. Eligibility criteria

The present research aimed to answer the following focused question: Is the low-level laser therapy effective to reduce the pain and accelerate the improvement of paresthesia after orthognathic surgery?

It was used the Population, Intervention, Comparison, Outcome and Study Design (PICOS) strategy to define the eligibility criteria, and the research question was based on the elements described at  Table 1.

**Table 1:**
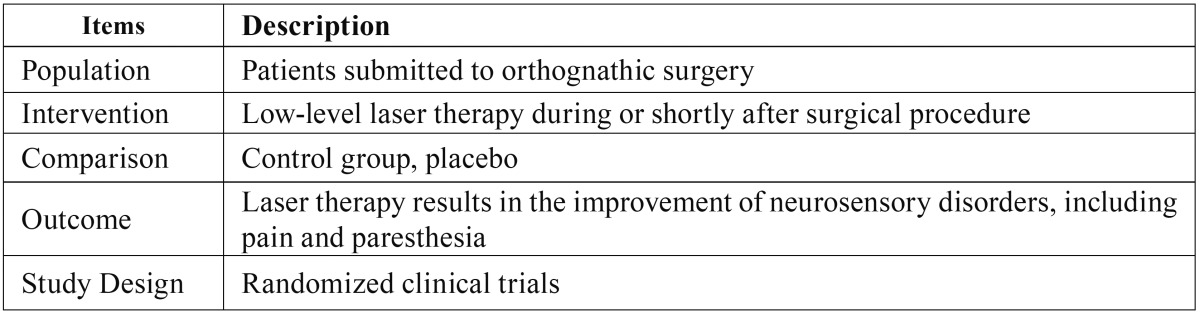
PICOS strategy adopted to achieve evidences in face of the research question.

Only randomized clinical trials were included. No language or publication year were imposed. It was excluded (1) studies from which it was impossible to extract data regarding at least one of the outcomes of interest; (2) abstracts or indexes; (3) letters to editors; (4) literature reviews; (5) medical glossaries; (6) protocols; (7) studies involving other surgeries; (8) prevalence studies; (9) studies that evaluated other parts of the body; (10) laboratory studies; (11) studies that did not use low-level laser; (12) book chapters; (13) case reports; and (14) studies in which laser therapy was not performed during or shortly after the surgical procedure.

3. Information sources

In order to identify relevant studies, a systematic search was conducted in the following electronic databases: PubMed, Scopus, Science Direct (only journals, excluding books and reference works), LILACS, SciELO, and Cochrane Central Register of Controlled Trials (CENTRAL). A grey literature search was performed through Google Scholar and OpenGrey to avoid potential selection bias. In addition, it was searched trials electronically at ClinicalTrials.gov.

4. Search

The Medical Subject Headings (MeSH) was used to select the descriptors. Boolean operators (OR and AND) were used to combine the descriptors. This search was performed in July 2016, and updated in November 2016. It was also conducted a hand search of cross-references from original articles to identify additional studies that could not be located in the electronic databases. The full electronic search strategy is illustrated in the  Table 2. All obtained references were exported to Mendeley™ Desktop 1.13.3 (Mendeley Ltd., London, England) software, in order to track potential duplicate records.

**Table 2:**
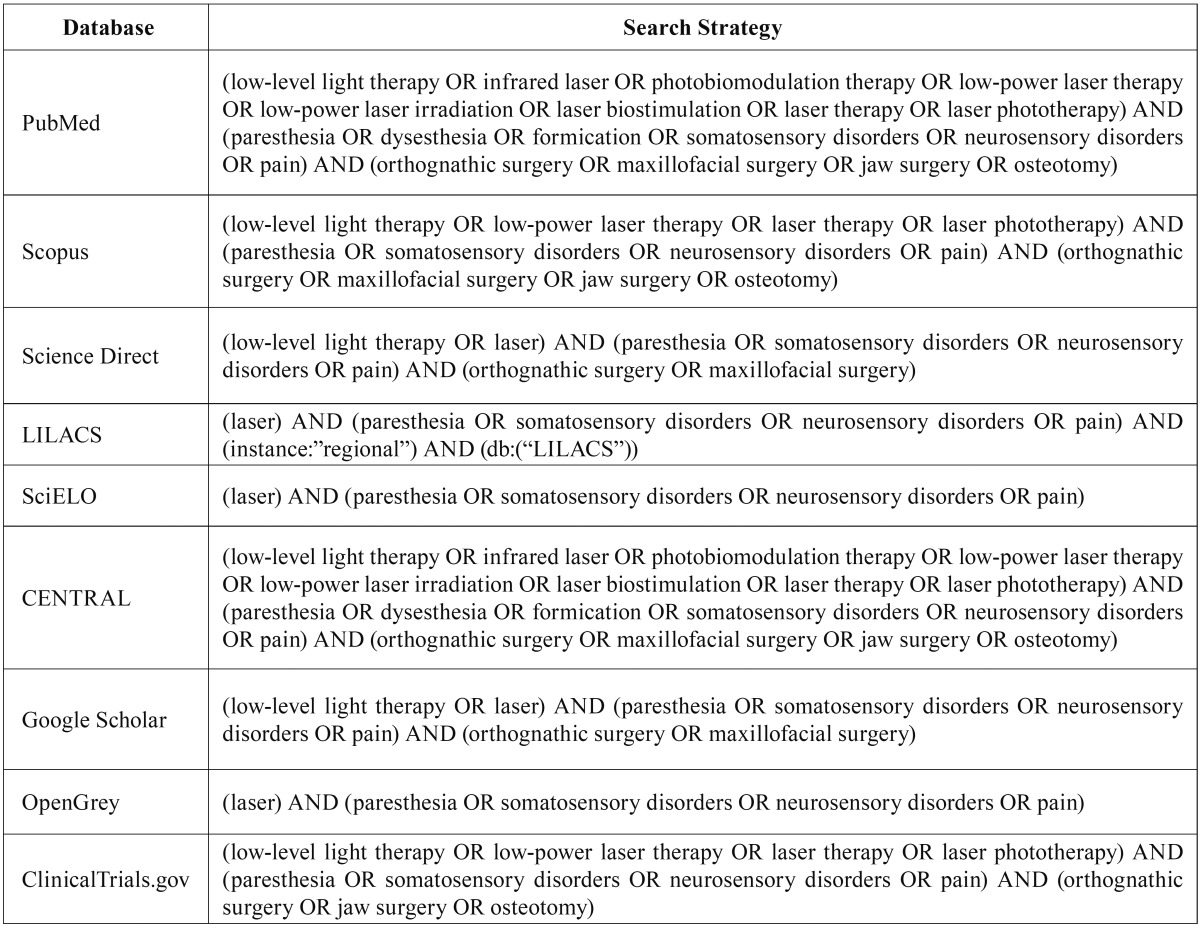
Electronic databases and applied search strategy.

5. Study selection

The data collection was independently performed by two reviewers (MAVB and LRP) who were not blinded for authorship information and journals’ names, in three different phases. First, titles were carefully read to exclude articles out of the scope of the research. At this stage, literature reviews, case reports, letters to the editor, laboratory studies, and others previously cited were also excluded. Then, in phase 2, abstracts of the remaining articles were read and inappropriate ones were excluded. The articles whose title or abstract did not present sufficient information were downloaded and had the full-text analyzed, in phase 3, in order to decide about their inclusion in the systematic review according to the eligibility criteria. In specific cases, when article did not present complete data, author was contacted by e-mail in order to obtain more information. When mutual agreement between the two reviewers was not reached, a third reviewer (PRSMF) was involved to make a final decision. Rejected studies and reasons for its exclusion were separately recorded.

6. Data collection process

One author (MAVB) collected the required information from the selected articles; a second author (LRP) cross-checked the in-formation to confirm the quality of the data extraction. Any disagreement was resolved by discussion with a third author (PRSMF). Attempts were made to contact the authors of the selected studies to retrieve missing information.

7. Data items

After filtration, full-text articles underwent data systematic extraction. Data was extracted regarding the study population (size, gender, and age), study design, characteristics of orthognathic surgery, low-level laser therapy protocol, including the region and time of application, assessment forms, and outcome measures, besides authorship, year of publication, and country of origin.

8. Risk of bias in individual studies

The risk of bias was assessed according to the Cochrane Guidelines for Clinical Trials (17). It was assessed six domains for evaluation, including the Selection Bias (random sequence generation and allocation concealment), Performance Bias (blinding of participants and personnel), Detection Bias (blinding of outcome assessment), Attrition Bias (incomplete outcome data), Reporting Bias (selective outcome reporting), and other potential sources of bias. It was rated the risk of bias as being low, unclear, or high according to established criteria.

9. Outcome measures

The reduction of postoperative pain, acceleration of recovery, and restoration of normal sensitivity in patients submitted to orthognathic surgery were considered the key findings. Outcome measures that evaluated the effectiveness of low-level laser therapy in reducing pain or improving sensitivity were considered.

10. Data Analysis

The heterogeneity among the included articles was analyzed through the examination of the study characteristics, such as dissimilarity between study participants, surgical procedures, laser protocol, and outcomes of interest (18). A meta-analysis was planned, considering that data from included articles were relatively homogeneous and appropriate for pooling. If the data were heterogeneous and inappropriate for a meta-analysis, a descriptive analysis and summary of the main findings of the selected studies was performed.

## Results

1. Study selection

**Figure 1:**
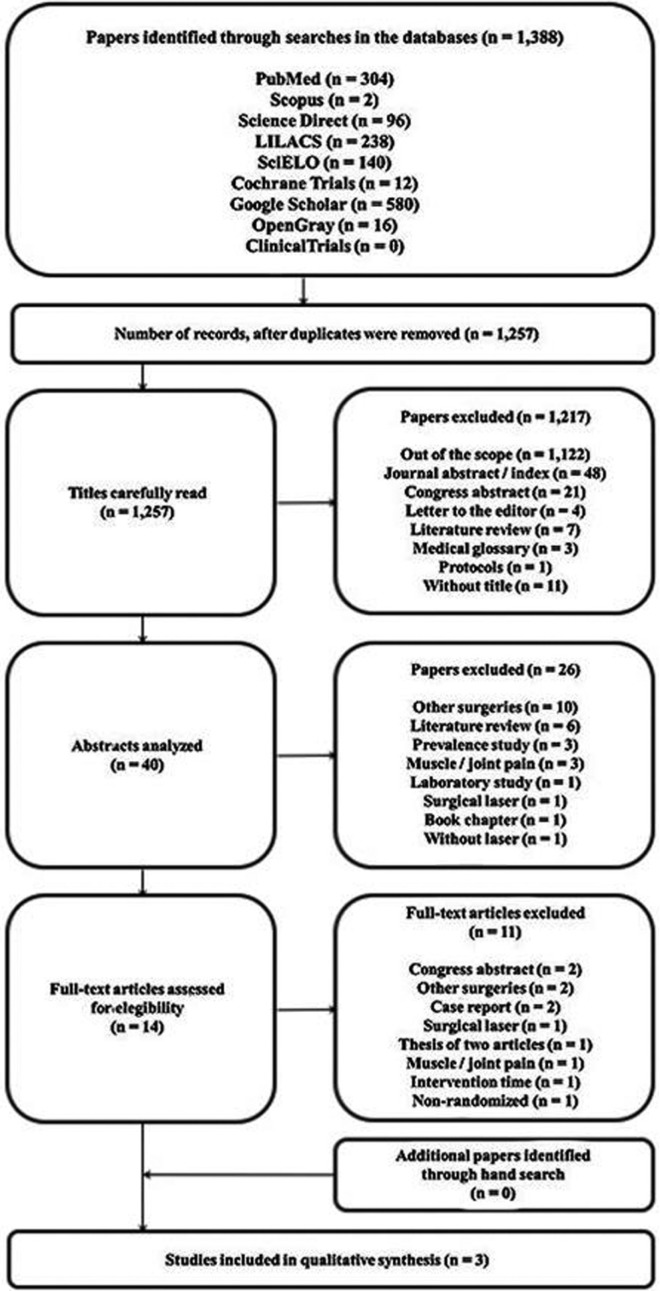
Flowchart showing the results of the search process.

The systematic search performed within eight electronic databases resulted in 1,388 references, of which 304 were collected from PubMed, 2 from Scopus, 96 from Science Direct, 238 from LILACS, 140 from SciELO, 12 from CENTRAL, 580 from Google Scholar, 16 from OpenGrey, and no article was collected from ClinicalTrials.gov. After removal of duplicate references, 1,257 titles were carefully read in phase 1, of which 1,217 were excluded. Then, in phase 2, a total of 40 abstracts were analyzed and 26 inappropriate references were also excluded. Therefore, a total of 14 studies were selected for analysis of the full text in phase 3. After reading of the full texts, only three articles (19-21) met the eligibility criteria and were included in the present systematic review. No additional study was included as a result of the hand search of cross-references. A flowchart depicting the selection process of references at each stage of the systematic review is provided in Figure 1.

2. Study characteristics

Three randomized clinical trials (19-21) were included in the present systematic review. Among these three articles, one addressed the reduction at the postoperative pain as the outcome of interest (19) and other two the improvement of paresthesia resulting from osteotomy (20,21).  Table 3 provides a summary of their characteristics. All articles were published in English, in 2014, two performed in Brazil and one in Chile. The studies were randomized double-blind clinical trials, two of them crossover (19,20) and the other controlled by a placebo group (21).

**Table 3:**
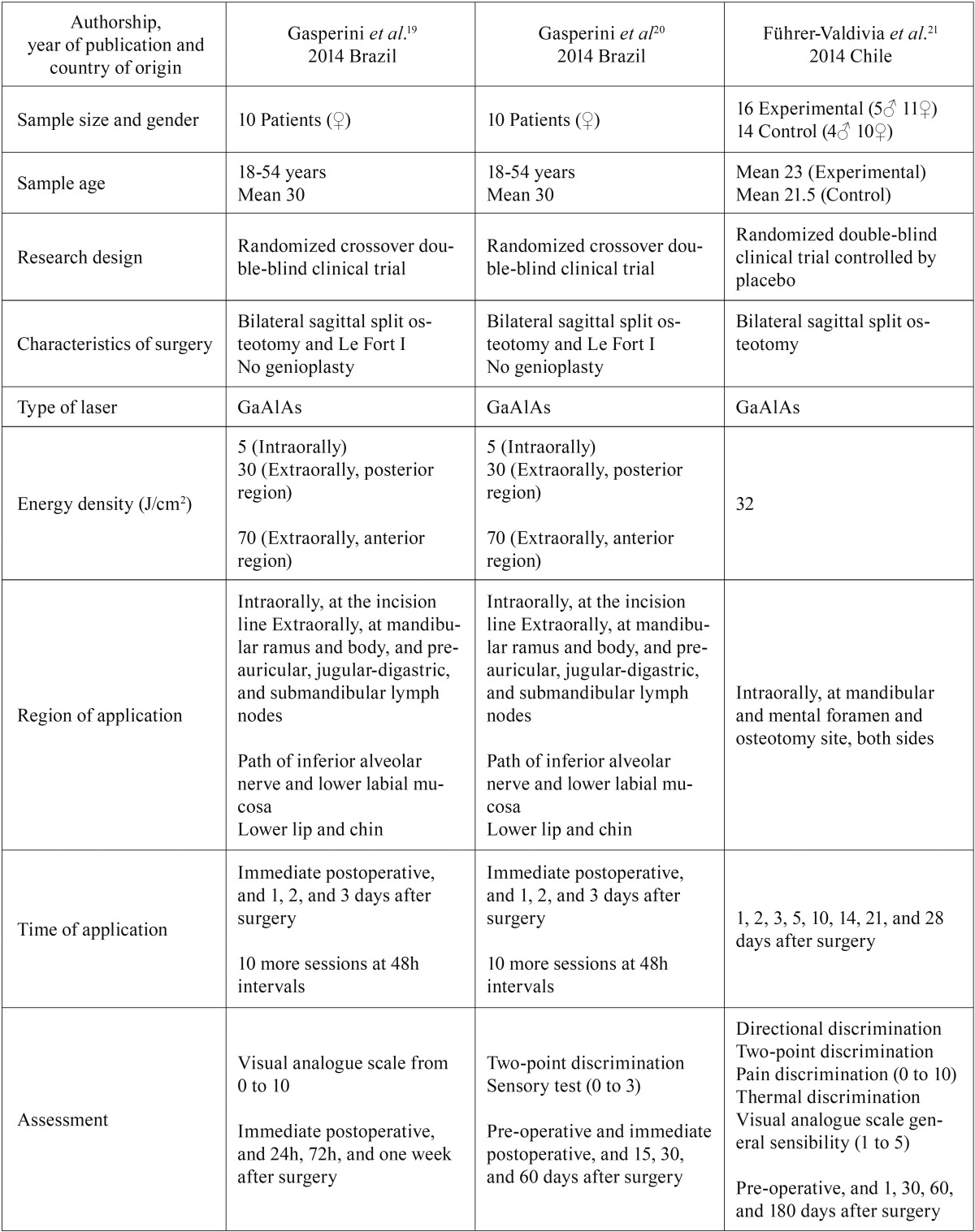
Characteristics of studies included in the systematic review.

Methodological characteristics of the two papers of Gasperini *et al.* (19,20) were the same in general. All subjects had undergone orthognathic bimaxillary surgery composed by Le Fort I and bilateral sagittal split osteotomies to correct dentofacial deformities. A GaAlAs low-level laser was used for therapy, with energy density varying between 5 J/cm2 for intraoral exposure and 30 J/cm2 or 70 J/cm2 for extraoral exposure. The surgical wound was exposed intraorally, and the mandibular ramus and body were exposed extraorally, immediately after and at 24, 48, and 72 hours after the surgery. After the fourth day, 10 additional applications, with an interval of 48 hours, were performed at the surgical wound on the path of the inferior alveolar nerve and the lower labial mucosa, intraorally, and the lower lip the chin region, extraorally. On the other side of the face, the laser unit was positioned at the same points but the laser was not activated. Assessment of post-operative symptoms varied according to the outcome of interest. For pain (19), a visual analogue scale (VAS) was used to measure its intensity immediately after the surgery and at 24 hours, 72 hours, and one week after the surgery. Subjects were asked about the degree of pain in each period using a scale from 0 (absence of pain) to 10 (maximum tolerable level of pain). For paresthesia (20), labiomental sensation was evaluated pre-operatively, immediately after the surgery and 15 days, 30 days, and 60 days after the surgery, by two-point discrimination and sensory tests. A 25x7 needle was used to determine the shortest distance that the patient could feel the two punctures and the same needle was used to stimulate labiomental region and the sensation was reported as a score from 0 to 3, with 3 being normal perception.

At the article of Führer-Valdivia *et al.* (21), all participants were intervened with a bilateral sagittal split osteotomy. For laser therapy, it was also used the GaAlAs low-level laser, but with an energy density of 32 J/cm2 for intraoral exposure. Both left and right mandibular and mental foramen and osteotomy site were exposed at 24, 48, and 72 hours and at days 5, 10, 14, 21, and 28 after surgery. The control group received the same laser application with laser light turned off, acting as a placebo. The outcome of interest was the improvement of paresthesia, assessed 24 hours and at 1, 2, and 6 months after surgery by means of five tests at the lower lip and 20 mm below oral commissure. Neurosensation was measured by dichotomous tests (tactile directional, two-point, pain and thermoalgesic discriminations) and ordinal test (VAS for sensitivity). For the directional discrimination, longitudinal movements with a nylon filament were performed without a logical sequence of 10 trials, and 7 correct answers indicated test as positive. For two-point discrimination, the distance between the points of a compass was stated at 15 mm, 10 mm and then joined and separated gradually up to 3 mm. Pain discrimination was tested by the same dry point compass, harmlessly punctured with the same pressure, assessed by means of a scale with 0, 2, 4, 6, 8, and 10, with 10 being maximum pain. Warm and cold materials were also applied at the sites of interest and sensations were related by patients. Finally, between five options, participants determined which score fitted more in relation to their personal perception of the affected areas, in a scale from 1 to 5, with 5 being normal perception.

The current medical literature does not standardize the anatomic region and the time of laser application. Therefore, it was observed great variations within the selected articles. Besides, the neurosensory evaluations were performed based on the results of different tests, also scored of different ways.

3. Risk of bias within studies

**Figure 2:**
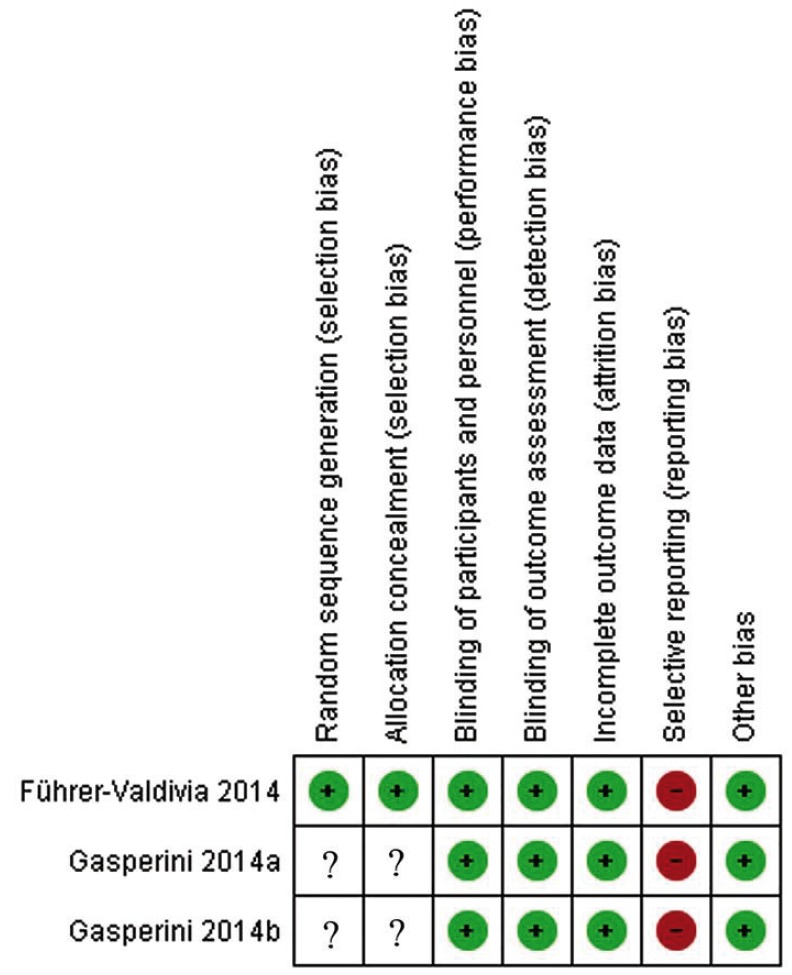
Risk of bias of the selected articles scored as (+) low risk, (-) high risk, and (?) unclear.

The methodological quality evaluation using the Cochrane Guidelines for Clinical Trials is shown in Figure 2. Gasperini *et al.*, in both articles (19,20), scored low risk of bias for most of the domains, having scored unclear for both selection bias, and high risk just for the reporting bias. Paper of Führer-Valdivia *et al.* (21) was classified as higher quality, having scored low risk of bias for almost all domains, except the reporting bias, also scored as high risk.

4. Results of individual studies

The effect of low-level laser therapy on the reduction of postoperative pain was one of the outcomes investigated. In this aspect, Gasperini *et al.* (19) showed no significant difference between irradiated and non-irradiated sides in the immediate postoperative assessment (*P*=0.442), but laser was effective to reduce pain at the 24 hours (*P*=0.007) and 72 hours (*P*=0.007) assessments.

The other investigated outcome was the improvement of paresthesia. At the study of Gasperini *et al.* (20), neurosensory recovery of the irradiated side of the patients was faster than that of the non-irradiated side. In the immediate post-operative period, both tests indicated no significant difference between the sides, but 15 days after surgery the sensitivity of the irradiated side had come back faster, assessed by the two-point discrimination (*P*=0.028) and sensory (*P*=0.005) tests. Differences between the two sides had increased gradually and remained significant 60 days after surgery, evaluated also by the two-point discrimination (*P*=0.005) and sensory (*P*=0.008) tests.

Führer-Valdivia *et al.*, at the other article (21), have used five tests to evaluate laser effectiveness on paresthesia. However, for directional, pain, and thermal discriminations tests, results were not favorable for any of the two groups. For general sensibility, results showed that low-level laser was effective for gradual improvement of paresthesia and, six months after surgery, participants reported 68.75% of recovery for laser group, compared with 21.43% for placebo (*P*=0.0095). Two-point discrimination test showed almost the same effectiveness, with 62.5% of patients of laser group presenting recovery of normal sensitivity (*P*=0.0631).

5. Synthesis of results and Risk of bias across studies

A meta-analysis was not possible. The use of laser in postoperative pain was evaluated in just one selected article (19), and the two studies included in this systematic review to evaluate the applicability of laser to improve the paresthesia after surgery (20,21) described different types of measures and different times of follow-up visits. Therefore, the pooled data from those studies were deemed not suitable because of the differences in the study designs and in the collected information.

## Discussion

This systematic review aimed to evaluate the effectiveness of low-level laser during or shortly after the surgical procedure in reducing postoperative pain or improving paresthesia in patients submitted to orthognathic surgery. Since this surgical procedure was introduced as a powerful method to correct dentofacial deformities, several strategies have been used as an attempt to control or decrease its potential complications or sequelae. Although it has been almost 50 years since Mester *et al.* (22) first demonstrated that laser phototherapy could relieve pain and promote tissue repair, its therapeutic value as a clinical armamentarium remains contentious and conclusions are yet to be fully confirmed.

Low-level laser therapy has been described in the literature as exerting a biomodulatory effect (10,11,23,24). Absorption of laser energy stimulates or inhibits enzymatic activities and photochemical reactions that induce cascades of reactions and physiological processes mediating inflammation and activating the immune system with broad therapeutic connotations (11). At their investigation, Gasperini *et al.* (19) showed that laser has important anti-inflammatory and analgesic actions, reducing pain on the irradiated side at the 24 and 72 hours postoperative evaluations. Immediately after surgery, it was not expected any reduction because there had been no time for laser biomodulation. One week later, no patient reported any pain on either side. However, the ideal protocol for laser utilization in oral surgeries has not yet been completely developed and this is probably the main reason for some contradictory studies found at the literature. Bjordal *et al.* (23) conducted a systematic review of randomized placebo-controlled trials to determine the mechanisms of action and clinical effects of laser therapy in acute pain, and observed a great amount of differences in methodologies and protocols.

The frequency of neurosensory impairment after orthognathic surgery is very high. According the systematic review performed by Colella *et al.* (25), the frequency assessed by objective methods is 63.3% while that obtained with subjective methods is 83% at the seventh postoperative day. Low-level laser irradiation on the affected innervation path has been shown to result in sensory improvement and seeks to accelerate recovery (10,13,26). Gasperini *et al.* (20) findings showed that there was an improvement in the sensitivity of the lower lip and chin in all patients on both the irradiated and non-irradiated sides, but on the treated side recovery was faster and was almost complete at the time of the last evaluation, 60 days after surgery. Führer-Valdivia *et al.* (21) reported differences between laser treated and non-treated patients, varying according to the tests used to perform the evaluation. The most expressive result was for general sensitivity assessed by the visual analog scale which demonstrated the normal recovery reached by a major number of patients from the laser group (68.75%) against just a few number of the control one (21.43%) six months after surgery. This suggests a beneficial effect of low-level laser therapy in neurosensory impairment of the lower third of the face after orthognathic surgery. However, as previously cited, the actual effect of the laser is difficult to quantify as varied greatly according to the used test.

Jenkins and Carrol (27) stated that most researchers frequently make critical errors and omissions when submitting papers for publication which makes reproducibility impossible. According to them, the most important beam parameters that should be reported are wavelength, power, irradiation time, beam area at the skin or surface, pulse parameters, anatomical location, number of treatments, and interval between treatments. In addition, more thorough reporting would include coherence, application technique (contact, projection, scanning, pressure), beam profile, and spectral width, as these may also be considered important. Then, authors should take care to measure and record these accurately before, during, and after an experiment or clinical trial. If there is no standardization in beam measurement, dose calculation and the reporting of these parameters, advancing the field of laser therapy will be more difficult.

## Conclusions

Individual studies suggested a positive effect of low-level laser therapy on the reduction of postoperative pain and acceleration of improvement of paresthesia related to orthognathic surgery. However, due to the insufficient number and heterogeneity of studies, a meta-analysis evaluating the outcomes of interest was not performed, and a pragmatic recommendation about the use of laser therapy is not possible. Further high-quality clinical trials are needed to increase the strength of evidence and to confirm the effectiveness of low-level laser for the treatment of neurosensory disorders after orthognathic surgery.
